# α-Asarone Attenuates Cognitive Deficit in a Pilocarpine-Induced Status Epilepticus Rat Model *via* a Decrease in the Nuclear Factor-κB Activation and Reduction in Microglia Neuroinflammation

**DOI:** 10.3389/fneur.2017.00661

**Published:** 2017-12-14

**Authors:** Hui-juan Liu, Xin Lai, Yan Xu, Jing-kun Miao, Chun Li, Jing-ying Liu, Yuan-yuan Hua, Qian Ma, Qixiong Chen

**Affiliations:** ^1^Chongqing Key Laboratory of Pediatrics, Chongqing, China; ^2^China International Science and Technology Cooperation Base of Child Development and Critical Disorders, Chongqing, China; ^3^Center for Clinical Molecular Medicine, Chongqing, China; ^4^Department of Neonatology, Children’s Hospital of Chongqing Medical University, Ministry of Education Key Laboratory of Child Development and Disorders, Chongqing, China

**Keywords:** α-asarone, epilepsy, microglia, inflammation, nuclear factor-κB

## Abstract

**Background:**

Temporal lobe epilepsy (TLE) is one of the most drug-resistant types of epilepsy with about 80% of TLE patients falling into this category. Increasing evidence suggests that neuroinflammation, which has a critical role in the epileptogenesis of TLE, is associated with microglial activation. Therefore, agents that act toward the alleviation in microglial activation and the attenuation of neuroinflammation are promising candidates to treat TLE. α-Asarone is a major active ingredient of the Acori Graminei Rhizoma used in Traditional Chinese Medicine, which has been used to improve various disease conditions including stroke and convulsions. In addition, an increasing number of studies suggested that α-asarone can attenuate microglia-mediated neuroinflammation. Thus, we hypothesized that α-asarone is a promising neuroprotective agent for the treatment of the TLE.

**Methods:**

The present study evaluated the therapeutic effects of α-asarone on microglia-mediated neuroinflammation and neuroprotection *in vitro* and *in vivo*, using an untreated control group, a status epilepticus (SE)-induced group, and an SE-induced α-asarone pretreated group. A pilocarpine-induced rat model of TLE was established to investigate the neuroprotective effects of α-asarone *in vivo*. For the *in vitro* study, lipopolysaccharide (LPS)-stimulated primary cultured microglial cells were used.

**Results:**

The results indicated that the brain microglial activation in the rats of the SE rat model led to important learning and memory deficit. Preventive treatment with α-asarone restrained microglial activation and reduced learning and memory deficit. In the *in vitro* studies, α-asarone significantly suppressed proinflammatory cytokine production in primary cultured microglial cells and attenuated the LPS-stimulated neuroinflammatory responses. Our mechanistic study revealed that α-asarone inhibited inflammatory processes by regulation the transcription levels of kappa-B, by blocking the degradation pathway of kappa B-alpha [inhibitor kappa B-alpha (IκB-α)] and kappa B-beta (IκB-β) kinase in both the SE rats and in primary cultured microglial cells.

**Conclusion:**

Taken together, these data demonstrate that α-asarone is a promising neuroprotective agent for the prevention and treatment of microglia-mediated neuroinflammatory conditions including TLE, for which further assessment studies are pertinent.

## Introduction

Epilepsy is a chronic brain disorder characterized by a recurrent predisposition for epileptic seizures, including status epilepticus (SE) crisis (1). The prevalence rate of epilepsy is about 7–14 per 1,000 people in developing countries, though often much higher in infants and elderly people (2, 3). Pharmacological intervention is the main strategy for treatment of epilepsy; however, approximately one-third of the epilepsy patients are drug-resistant (4). Furthermore, currently available antiepileptic drugs (AEDs) can merely suppress seizure symptoms and are ineffective at preventing the occurrence of the underlying pathology of epileptogenesis (5, 6). Thus, the development of effective therapies that target the molecular and cellular mechanisms underlying the epileptogenic process is urgently required.

Temporal lobe epilepsy (TLE) is a type of drug non-responsive epilepsy, with about 80% of the TLE patients falling into this category (7). Various clinical, pathological and physiological studies strongly suggested that the inflammatory processes in the brain play an important role in the epileptogenesis (8–12). For example, it has been shown that the immune system is activated in the brain of patients with TLE and that immune therapies are often more effective than AEDs administration in the treatment of TLE (9). These findings had also been observed in an experimental model of TLE in rats (13).

Microglia, the resident immune cells of the central nervous system (CNS) play an important role in neuroinflammation. Previous studies have shown that microglia were extensively activated in the brain tissues of drug-refractory epileptic patients and of epilepsy-induced animal models (14, 15), whereas the alleviation in microglia activation reduced excitotoxin-induced brain damage (16). In the SE brain, activated microglia not only could increase the level of cytokines [including interleukin-1β (IL-1β) and tumor necrosis factor-α (TNF-α)], but also could induce enzyme activity [including nitric oxide synthase (iNOS) and cyclooxygenase-2 (COX-2)], which subsequently increased neuronal excitability and seizure susceptibility (17). In addition, activated microglia also triggered a cascade of downstream inflammatory events by activating nuclear factor-κB (NF-κB), which is known to play an important role in the regulation of inflammation response (18, 19).

Alpha (α)-asarone (1-propenyl-2,4,5-methoxybenzyl) is the major active ingredient of Acori Graminei Rhizoma, a Traditional Chinese Medicine used for the treatment of stroke and convulsions (20, 21). Recent reports have shown that α-asarone might exert anti-inflammatory effects (22), and subsequently alleviate epilepsy by modulating γ-aminobutyric acid (GABA) receptors (23). Consistent with various experimental models of epilepsy, our recent report studied the antiepileptic effects of α-asarone (24). However, to date, the mechanisms underlying the beneficial effect of α-asarone on TLE are still unclear. More recently, several studies have shown that α-asarone could dramatically decrease the activation of microglia, and reduce the levels of cytokines (including IL-1β and TNF-α) and the level of enzyme activation (including iNOS and COX-2) (25, 26). α-Asarone attenuates microglia-mediated neuroinflammation *via* the decrease in the levels of NF-κB activation by blocking the signaling pathway of kappa B-alpha (IkB-α) degradation (27). Thus, we propose that α-asarone may be a potential therapeutic agent against epilepsy *via* its effect on the alleviation of microglia activation and/or its anti-inflammatory effects. In the present study, we investigated this hypothesis using an *in vivo* lithium-pilocarpine-induced SE model in rats and an *in vitro* primary cultured microglia cell model.

## Materials and Methods

### Reagents and Antibodies

α-Asarone, lipopolysaccharide (LPS), lithium chloride, pilocarpine, methylscopolamine, and 3-(4,5-dimethylimidazole-2-yl)-2,5-diphenyl-tetrazolium bromide (MTT) were provided by Sigma-Aldrich (St. Louis, MO, USA). Phosphatase inhibitor and protease inhibitor cocktail tablets were purchased from Roche (Indianapolis, IN, USA). Dulbecco’s modified Eagle’s medium (DMEM) and fetal bovine serum (FBS) were purchased from Gibco-BRL Technologies (Carlsbad, CA, USA). NF-κB pathway sampler kit was purchased from Cell Signaling Technology (Danvers, MA, USA).

### Animals

Adult male Sprague-Dawley (SD) rats weighing 200–250 g were supplied by Chongqing Medical University (Chongqing, China). The rats were housed under controlled standard conditions (23 ± 2°C; 55 ± 20% humidity; 12:12 light/dark cycle with lights on at 7 a.m.). Food and water were available *ad libitum*. All efforts were made to minimize animal suffering and reduce the number of animals used in the experiments. All experiments were performed in accordance with the rules established by the Experimental Animal Administration Committee of the University and with the National Institutes of Guide for Care and Use of Laboratory Animals (NIH Publications no. 80-23, revised 1985).

### Establishment of the SE Model

All SD rats were divided randomly into the control group, the SE-induced group (SE group) and SE-induced α-asarone pretreated group (SE-asarone group). The SE and the SE-asarone group rats were injected with lithium chloride (127 mg/kg of body weight, i.p.). Methylscopolamine bromide (1 mg/kg of body weight, i.p.) was administered approximately 20 h later to limit the peripheral effects of pilocarpine. Pilocarpine hydrochloride (40 mg/kg of body weight, i.p.) was injected 30 min after methylscopolamine administration. Seizure activity associated with SE commenced 20–40 min after pilocarpine administration. Behavioral changes were recorded and graded according to the Racine’s scale (28). To improve survival, diazepam (DZP, 2 mg/kg of body weight, Hubei, China) was injected 2 h after SE induction. The rats in the SE-asarone group were administered α-asarone (100 mg/kg, i.p.) 1 h before administration of pilocarpine as described by Chen et al. (24). On the other hand, the rats in the control group were injected with lithium-methylscopolamine followed by saline as a substitute to pilocarpine. These rats also were administered with the same dose of DZP injection after saline injection 2 h, similarly to the other two groups. All SD rats were injected with 0.9% saline (10 ml, twice a day, i.p.) as well as fed water-soaked feed until they were able to eat normal dry food pellets (29) to restore volume loss associated with SE. SD rats which did not develop class 4 or 5 seizures according to Racine’s scale or died were excluded from follow up analysis.

### Spatial Memory Performance Assessments

Spatial learning and memory were assessed using the Morris water maze (MWM) task, 4 weeks after pilocarpine treatment. The MWM apparatus consists of a black circular pool (180 cm diameter, 45 cm high) filled with water (30 cm depth) at 26 ± 1°C. The pool is virtually divided into four equivalent quadrants, with a submerged escape platform (12 cm diameter) placed in the middle of one of the quadrants, equidistantly from the sidewalls, and the center of the pool, 1 cm below the water surface. Styrofoam pieces are afloat on the water to make the platform invisible. All rats were trained to escape the water by finding the platform in a series of four training sessions in 5 consecutive days and a final test was performed 24 h after the last training session. For each training session, all rats were randomly placed into one of the four quadrants facing the wall by a blinded observer. Each rat was given 120 s to find and mount the platform. The training session was considered finished after the rat successfully reached and climbed onto the hidden platform, where it was allowed to rest for 20 s. For each rat, the escape latency was recorded. Alternatively, the trial was also terminated if the rat failed to reach and/or climb onto the platform within the 120 s. In these cases, the blinded observer rescued the rat onto the platform an escape latency of 120 s was recorded. One day after the last training session, a probe trial was conducted in which the escape platform was removed from the pool. Each rat was allowed to swim for 120 s and the time spent in each quadrant was recorded.

### Behavioral Recording

Seven days after SE inducement, all rats were video monitored daily from 8 a.m. to 18 p.m. for a 4-week period, in order to record the occurrences of spontaneous recurrent seizures (SRS). The severity of the seizures was assessed using the Racines scale and the Veliskova scoring system (28, 30). The latency period (days), seizure frequency (times per day), and behavioral score were also recorded. All video recordings were done during the light period, only Racines’ class 4 or 5 seizures were analyzed. The recordings were analyzed by two experimental observers who were blind to group allocation.

### High-Performance Liquid Chromatography-Tandem Mass Spectrometry Assay

Three days after SE, brain tissues of five rats were grinded with 1 ml/g 0.9% NaCl. Subsequently, 0.5 ml homogenate was taken and extracted by 1.5 ml methyl alcohol, then the brain tissue extractions were dried by nitrogen at 40°C, the dried powders were dissolved to solutions by methyl alcohol (internal standard by 10 µg/ml 4-methoxybenzophenone). After that, the solutions were centrifugated and the liquid supernatants were finally retained. Meanwhile, α-asarone solutions with concentrations of 0.4, 4, 8, 16, 32, and 64 µg/ml (dissolved by methyl alcohol and internal standard by 10 µg/ml 4-methoxybenzophenone) were also prepared. The solutions were analyzed by high performance liquid chromatography-tandem mass spectrometry (HPLC-MS/MS, HP110, Shanghai, China), each peak area of α-asarone was recorded. Linear regression of peak area vs. concentration was conducted, and the concentration of α-asarone in the brain was calculated by the regression equation.

### Primary Culture of Microglia

Microglial cells were isolated from cultures of newborn rat brains according to a method previously described (31, 32), with minor modifications. Briefly, cerebral cortices were isolated from 1 to 2 days old SD rats, stripped of the meninges, dissociated by trituration, and digested in the presence of 0.25% trypsin for 30 min at 37°C. The cortical fragments were resuspended in DMEM containing 10% FBS, 100 IU/ml penicillin, and 100 mg/ml streptomycin. Fragments were made into single cell suspensions by repeated pipetting. The cell suspensions were plated in 75 cm^2^ flasks at a density of 4 × 10^6^/ml at 37°C under a humidified 5% CO_2_ and 95% air atmosphere for 10–14 days with medium replaced every 3 days. Microglia were harvested by gentle shaking at 37°C for 4 h. Cultures with >95% purity of microglia (identified by a CD-68-specific antibody) were used for follow-up experiments.

### Cell Viability Assay

Cell viability was determined by the salt 3-[4,5-dimethylthiazol-2-yl]-2, 5-diphenylα-asaronerazolium bromide (MTT) assay. Briefly, microglial cells were plated into 96-well plates at a density of 1 × 10^5^ cells per well for 24 h and incubated with various concentrations of α-asarone (25, 50, and 100 µg/ml) for 1 h before treatment with LPS (1 µg/ml) for another 24 h. Then the cells were incubated with MTT (0.5 mg/ml added to each well), 4 h at 37°C, 5% CO_2_. After this incubation period, the supernatant was removed from each well; the colored formazan crystal produced from MTT was dissolved in 0.15 ml DMSO, The absorbance values were measured at 570 nm using a multiscanner autoreader.

### Electrophoretic Mobility Shift Assay

The electrophoresis mobility shift assay (EMSA) for nucleoprotein extracts (10 µg) was performed using the Odyssey Infrared EMSA Kit (LI-COR, USA) according to the manufacturer’s instructions (33). Two oligonucleotides probes (wild-type NF-κB consensus-forward and repetitive SET NF-κB-forward) were labeled with IR700 dye (Bioneer Corporation, USA) and annealed to generate double-stranded probes at a final concentration of 0.01 pmol/μl. The binding reactions were incubated with NF-κB specific probe or mutant probe for 20 min at room temperature, and the mixture were isolated in a 4% Tris-glycine-EDTA gel for 70 min run at 70 V in darkness. Then the gel was scanned using the LI-COR Odyssey system (LI-COR, USA) at a wavelength of 700 nm. The wild-type NF-κB consensus oligonucleotides (forward: agttgaggggactttcccaggc and reverse: gcctgggaaagtcccctcaact) and mutant NF-κB consensus oligonucleotides (forward: agttgaggccactttcccaggc and reverse: gcctgggaaagtggcctcaact) probes were a gift from Dr. Song Weihong (Chongqing Medical University, China).

### Enzyme-Linked Immunosorbent (ELISA) Assay

The content levels of TNF-α and of IL-1β in the brain tissue supernatant of rats (*n* = 5 at each time point) and in the protein lysate of cultured microglia from the control and the SE and SE-asarone groups (*in vivo* and *in vitro*) were determined with ELISA kit (USCN, Wuhan, China), according the manufacturers’ to instructions. Total protein samples were extracted and total protein concentration was determined using an enhanced BCA Protein Assay kit (Beyotime, Harman, China) before the ELISA. Protein concentration measures obtained by ELISA were in assessed in duplicates and normalized using the samples initial total protein concentration. The results were expressed as pg/mg of protein.

### Western Blot Analysis

Total protein was extracted from the hippocampus and the adjacent cortex as well as from cultured microglia with RIPA Lysis Buffer (Beyotime, Harman, China) containing protease inhibitors. A total of 30 µg of total protein was loaded onto the sodium dodecyl sulfate-polyacrylamide gel and separated by electrophoresis (Bio-Rad, CA, USA). The protein bands were transferred onto 0.20 µm polyvinylidene difluoride (PVDF) membranes (Bio-Rad, CA, USA) and then probed with different primary antibodies: p-IKK (1:500), IKK (1:1,000), p-IκB-α (1:500), inhibitor kappa B-alpha (IκB-α, 1:1,000), iNOS (1:500), and COX-2 (1:500) (CST, USA). The PVDF membrane was incubated with the respective antibodies overnight at 4°C. Subsequently, the membranes were incubated with the appropriate secondary antibodies for 1 h at room temperature after washings with Tris-buffered saline with Tween (Sigma-Aldrich, USA). As a protein loading control, the membranes were incubated with monoclonal rat anti-GAPDH (1:10,000) (Sigma-Aldrich, USA). Immunoreactive bands were visualized by enhanced chemiluminescence (Bio-Rad, USA).

### Immunohistochemistry

At various time-points after SE induction, the rats were deeply anesthetized with pentobarbital sodium (50 mg/kg) and heart perfused with ice-cold 0.9% NaCl followed by chilled 4% paraformaldehyde in 0.01 M PBS as described previously (34). After perfusion, brains were removed promptly and postfixed (paraformaldehyde) for 24 h at 4°C. After overnight incubation in a solution of 30% sucrose in 0.1 M PBS for cryoprotection, transverse 30 µm thickness sections were cut from frozen blocks with a sliding microtome. Serial coronal sections were cut through the dorsal-ventral extension of the hippocampus (at a level corresponding to 2.8–4.5 from Bregma). Every sixth section of the hippocampus was selected for quantitative immunohistochemical analysis as previously described (35).

Immunohistochemical staining was performed using the avidin–biotin peroxidase complex detection kits (Zhongshan Golden Bridge, Beijing, China) according to the manufacturer’s instructions. Sections were incubated with CD-68 (Abcam, USA) overnight at 4°C. Negative control samples were also prepared by incubating the sections with PBS without antibodies and appropriate avidin–biotin complex solutions at 37°C for 20 min for IHC detection.

Images of the hippocampal CA1, CA3, and the adjacent cortex areas were captured using an OLYMPUS PM20 automatic microscope (Olympus, Tokyo, Japan). Cells were viewed with a 100× objective. A blinded operator oblivious to the identity of the section, assessed the total number of CD-68 cells stained in the predefined areas (400 μm × 400 μm) using an image analysis system (Image-Pro Plus Media Cybernetics, Silver Spring, MD, USA) to generate an automated cell count. An average of 10 sections per rat were analyzed. In all regions, cells which were morphologically intact and clearly identifiable were counted. The cell values of counted in the right and left hemispheres of the section were averaged as there was no significant difference in cell configuration between the two hemispheres. The number of positive cells in 10 sections was averaged to obtain a mean value for each animal. Two observers blinded to group allocation collected the data.

### Statistical Analysis

All data were presented as the mean ± SE for each independent experiment. Differences between groups and conditions were assessed using the Tukey multiple comparison test. One-way analysis of variance (ANOVA) was used to detect differences between groups. All statistical analyses were performed using the software SPSS 19.0, and the level of significance was set at *p* < 0.05.

## Results

### Effect of α-Asarone on SE-Induced Cognitive Function

**Figure 1:**
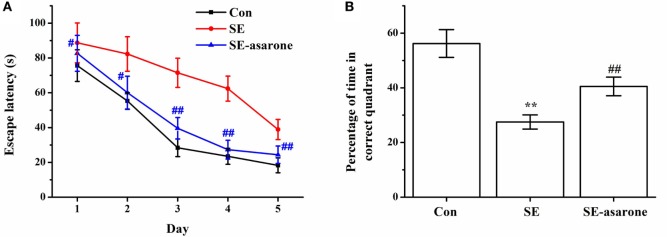
Effect of α-asarone on SE-induced learning and memory impairment, as assessed the by water maze test, 4 weeks after Li-pilocarpine administration. Escape latency to the platform in 5 days **(A)**, percentage of time in the correct quadrant **(B)**. Con, control group; SE, status epilepticus model group; SE-asarone, pretreatment with α-asarone group before SE induction. *N* = 10. **p < 0.01, vs. Con group; ^#^*p* < 0.05, ^##^*p* < 0.01, vs. SE group.

The effect of α-asarone on the SE-induced cognitive function was evaluated using a water maze test 4 weeks after the pilocarpine injection. Results showed that the escape latency of the SE-induced rats was significantly higher than that of the saline-treated rats (control group), whereas pretreatment with α-asarone (SE-asarone, 100 mg/kg, i.p.) dramatically shortened escape latency, from the first training session (Figure 1A). We normalized escape latency values of each training session against the escape latency values observed on the first training session. Twenty-four hours after the last learning trial, a probe test where the submerged platform had been removed from the pool, was performed. The results showed that the SE rats pretreated with α-asarone spent much more time (SE-asarone, 40.5 ± 3.4 s) in the target quadrant (where the submerged platform had been placed during the training sessions) than the rats in the SE group (27.5 ± 2.6 s) (Figure 1B). These results indicate that α-asarone significantly improved the cognitive impairment induced by SE.

### Effect of α-Asarone on the Characteristics of SRSs after SE

**Figure 2:**
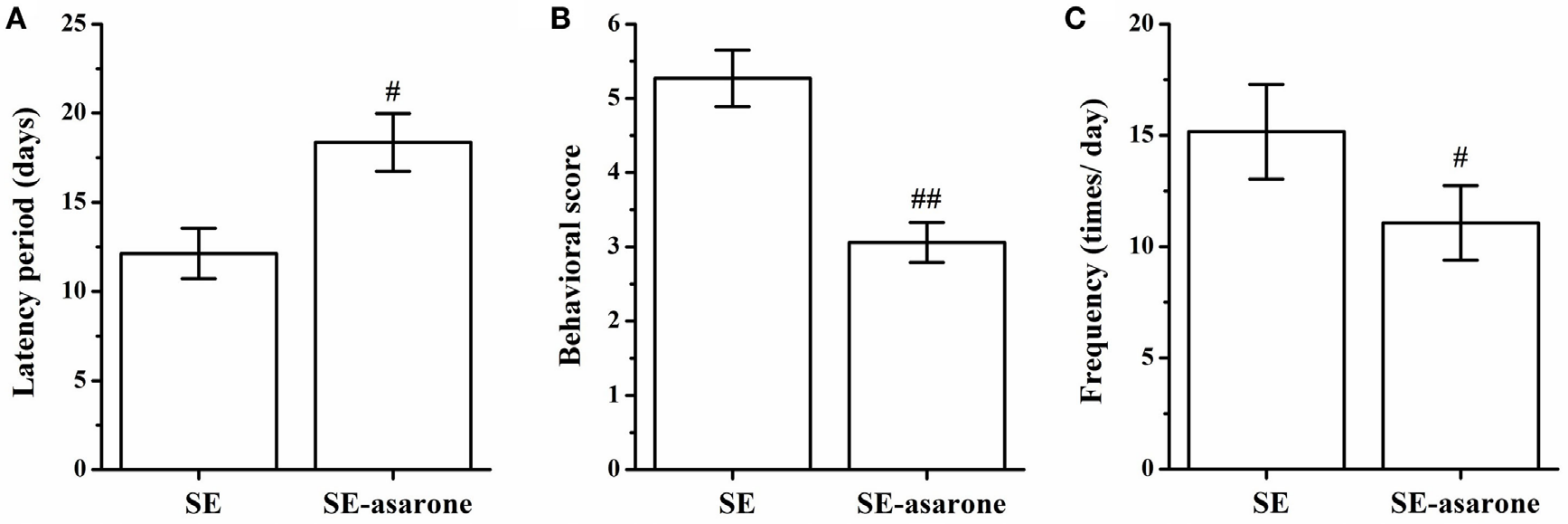
Effect of α-asarone on the characteristics of SRSs after SE inducement. All the rats were monitored to record SRSs. α-asarone had a significant effect on characteristics of SRSs after SE. Results for latency **(A)**, behavioral score **(B)** and frequency **(C)** between groups. SE, status epilepticus model group; SE-asarone group, pretreatment with α-asarone group before SE induction, *n* = 10. ^#^*p*<0.05, ^# #^*p* < 0.01, vs. SE group.

All the rats were monitored to record SRSs, and no seizures were recorded at any time in the control group. The latency, frequency and behavioral score of SRSs were recorded in the SE group and in the SE-asarone groups. As shown in Figure 2, the average latency of seizure was 12.13 ± 1.41 s in the SE group and 18.36 ± 1.62 s in the SE-asarone group, respectively (Figure 2A). Compared with the SE group, pretreatment with α-asarone significantly reduced the behavioral score (5.27 ± 0.38 and 3.06 ± 0.27 in the SE and SE-asarone group, respectively, Figure 2B) and the frequency of seizures (15.16 ± 2.13 and 11.07 ± 1.67 times per day in the SE and SE-asarone group, respectively, Figure 2C). Together, these results indicate that α-asarone dramatically alleviate epilepsy.

### Effect of α-Asarone on the Activation of Microglia Following SE

**Figure 3:**
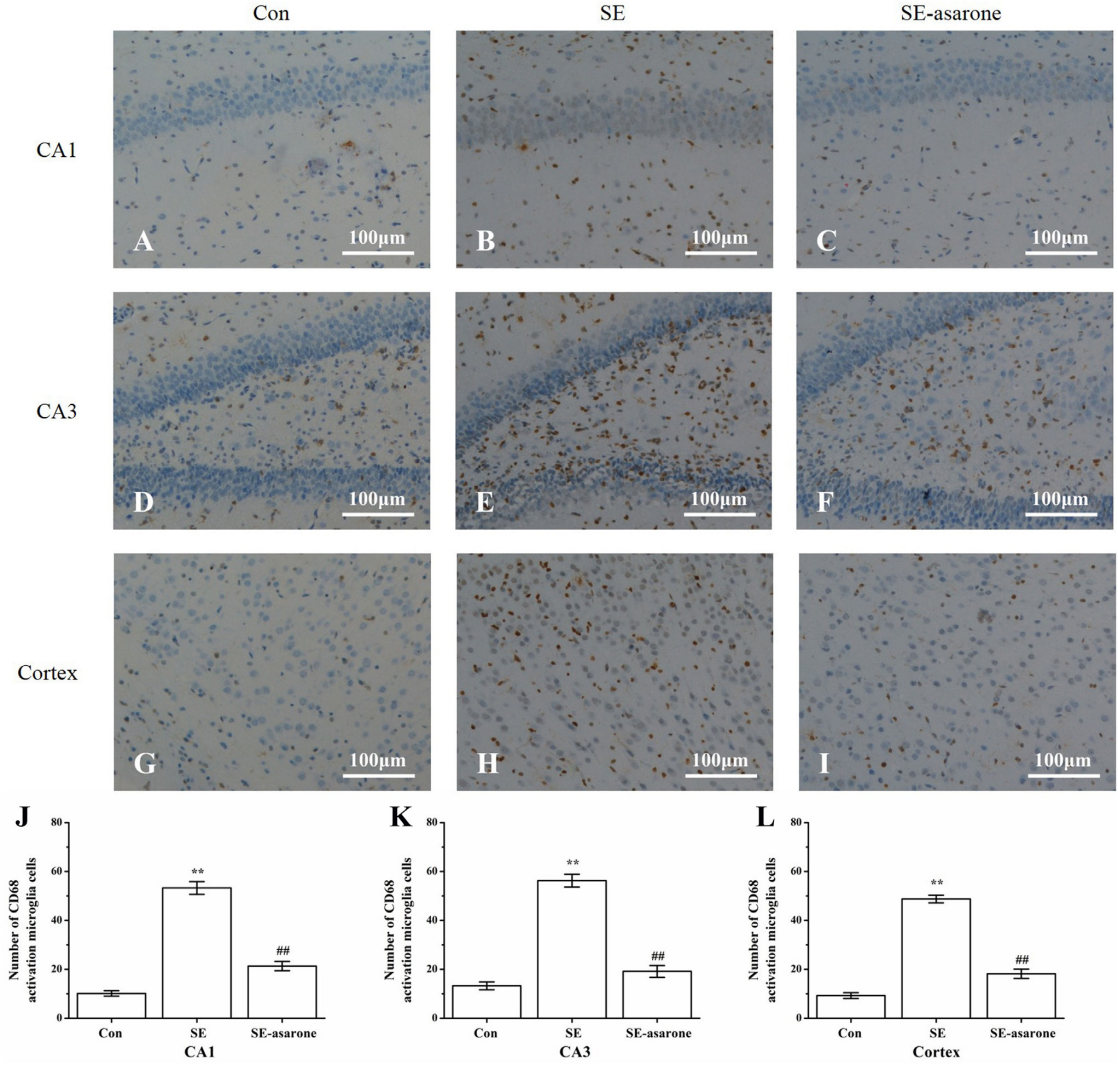
The effect of α-asarone on activation of microglia after SE induction. CD68-immunopositive microglia in the hippocampal CA1, CA3, and the adjacent cortex following SE were counted by IHC. Representational images of the CD68-immunopositive cells in the hippocampal CA1 **(A–C)**, CA3 **(D–F)**, and in the adjacent cortex **(G–I)** of each experimental rat group. Quantitative analysis of CD68-immunopositive microglia in the predefined areas of the hippocampal CA1 **(J)**, CA3 **(K)**, and the adjacent cortex **(L)**. Con, control group; SE, status epilepticus model group; SE-asarone, pretreatment with α-asarone group before SE induction; Scale bar = 100 µm. *n* = 5. *p < 0.05, **p < 0.01, vs. Con group; ^#^ *p* < 0.05, ^# #^*p* < 0.01, vs. SE group.

To determine the influence of SE on the activation of microglia in the hippocampal CA1, CA3, and the adjacent cortex, we used microglia marker CD-68 to stain microglia in all groups at 3 days after SE induction. Results showed that CD-68-positive cells significantly increased in the SE group (Figures 3B,E,H) compared to the control group (Figures 3A,D,G), whereas pretreatment with α-asarone before SE induction (Figures 3C,F,I) dramatically decreased CD-68-positive cells, even though the number of CD-68-positive cells in this group was still much higher than that of the control group. These results indicate that pretreatment with α-asarone greatly alleviated the activation of microglia after SE induction in the predefined areas of the hippocampal CA1, CA3 and the adjacent cortex (Figures 3J–L).

### α-Asarone Suppressed Activation of NF-κB in the Brain Tissue following SE Induction

**Figure 4:**
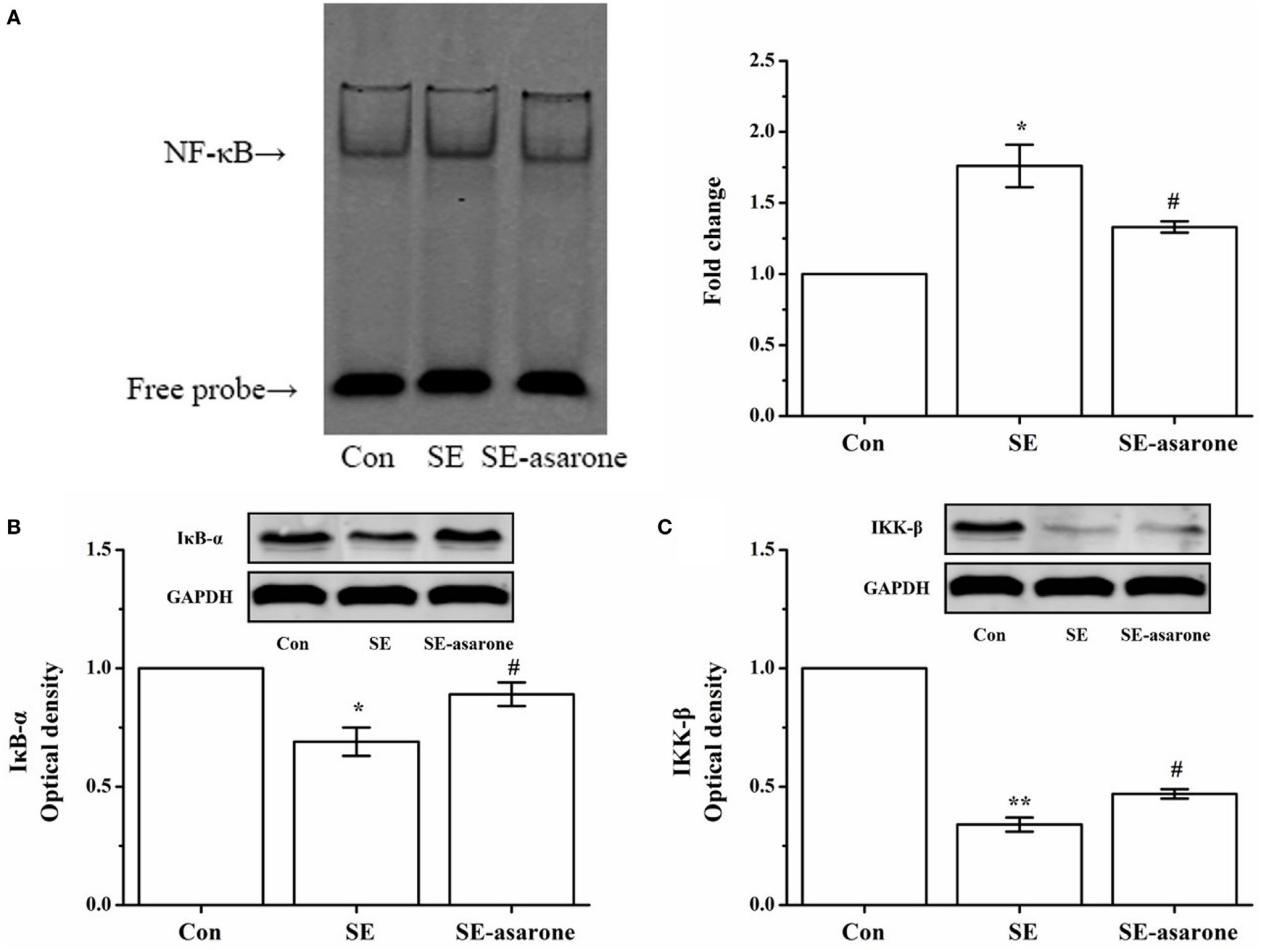
The effect of α-asarone on the activation status of NF-κB, degradation of IκB-α, p-IκB-α, and reduction of IKK-β and p-IKK-β after SE induction. EMSA analysis for NF-κB nuclear translocation **(A)**, western blot for IκB-α **(B)** and IKK-β **(C)**. The quantitative data are presented on each panel, respectively. Protein expression of GAPDH was used as the internal control for Western bloat protein loading. Con, control group; SE, status epilepticus model group; SE-asarone, pretreatment with α-asarone group before SE induction; NF-κB, nuclear factor-κB; EMSA, electrophoresis mobility shift assay; IκB-α, inhibitor kappa B-alpha; IKK-β, I kappaB kinase-β. *x* ± s, *n* = 5. *p < 0.05, **p < 0.01, vs. Con group; ^#^*p* < 0.05, ^##^*p* < 0.01, vs. SE group.

The NF-κB pathway activation requires degradation of IκB-α, I kappaB kinase-β (IKK-β), and a subsequent nuclear translocation of the free NF-κB (p65). Here, the free NF-κB activates of genes with NF-κB binding sites. Therefore, the effect of α-asarone on the activation of NF-κB pathway in the hippocampus and cerebral cortex following SE induction was investigated by EMSA and Western blot analysis. Our results show an increase in degradation of IκB-α, IKK-β after SE induction, following by translocation of the NF-κB p65 subunit to the nucleus. Pretreatment with α-asarone restrained IκB-α, IKK-β degradation, thus blocking the translocation of the NF-κB p65 subunit to the nucleus (Figure 4).

### Effect of α-Asarone on iNOS and COX-2 Protein Expression in Pilocarpine-Induced Rat

**Figure 5:**
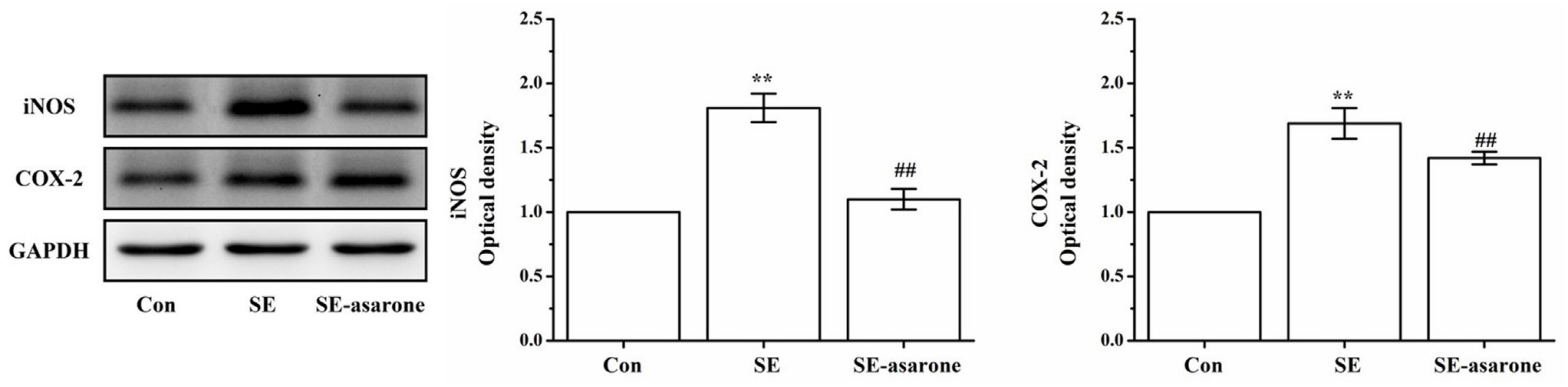
The effect of α-asarone on iNOS and COX-2 protein expression levels. Western blot analysis of iNOS and COX-2 expression. The quantitative data are displayed on each panel, respectively. Protein expression of GAPDH was used as the internal control for Western bloat protein loading. Con, control group; SE, status epilepticus model group; SE-asarone, pretreatment with α-asarone group before SE induction; iNOS, including nitric oxide synthase; COX-2, cyclooxygenase-2. *x* ± s, *n* = 5. *p < 0.05, **p < 0.01, vs. Con group; ^#^*p* < 0.05, ^##^*p* < 0.01, vs. SE group.

The iNOS and COX-2 protein expression were assessed in the brain tissue at 3 days after SE induction by Western blot. Our results show that iNOS and COX-2 protein expression in the SE group were significantly higher than those of the control group. Levels of iNOS and COX-2 protein expression in the SE group pretreatment with α-asarone were significantly lower than those of the SE group, though still much higher than those of the control group (Figure 5). These results indicate that α-asarone might significantly decrease iNOS and COX-2 expression after SE induction.

### α-Asarone Suppresses Pilocarpine-Induced Proinflammatory Cytokine Production

**Figure 6:**
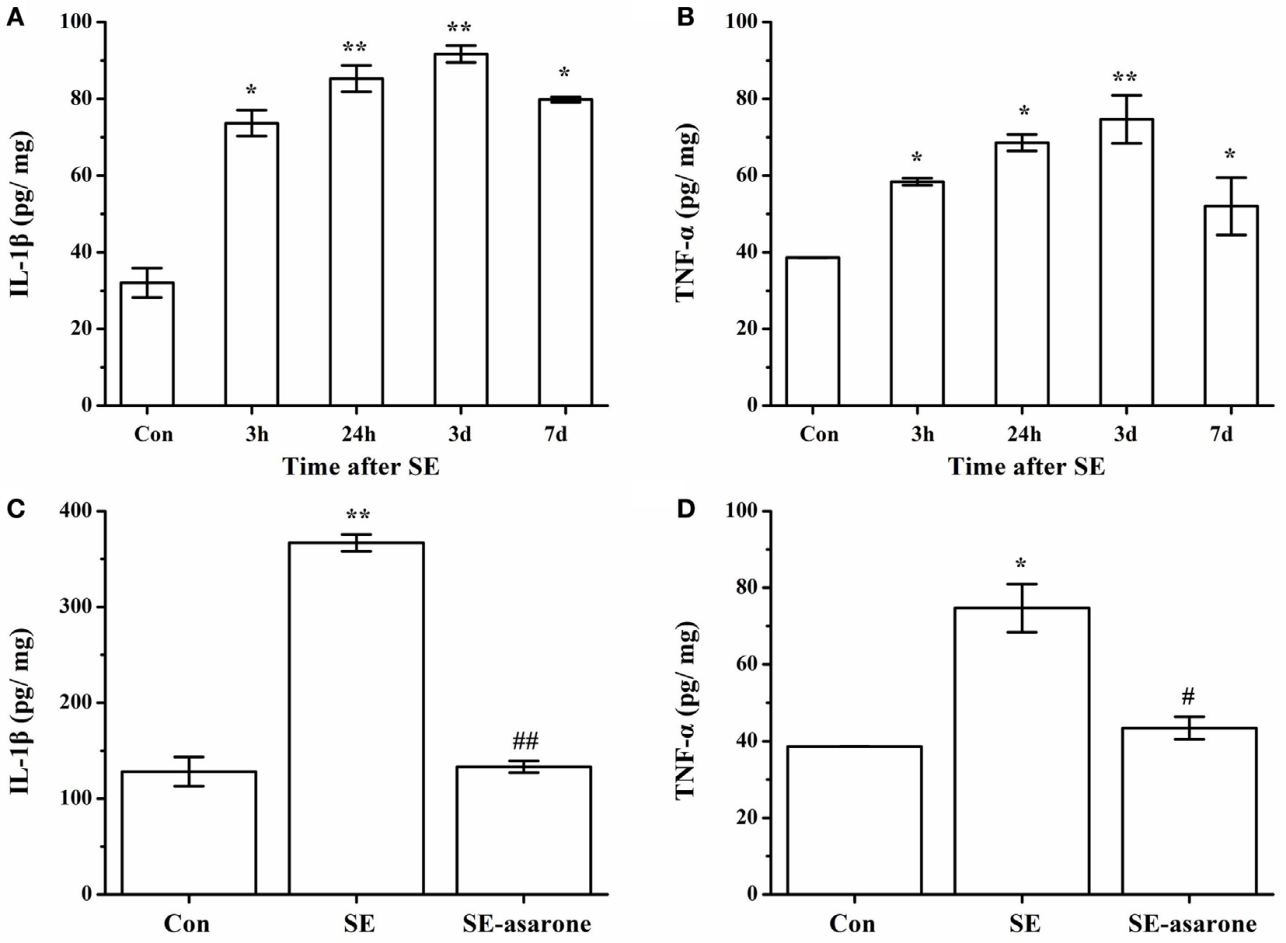
The effect of α-asarone on IL-1β and TNF-α concentration between the groups: ELISA analysis of the IL-1β, TNF-α protein expression levels in the SE group and in the α-asarone pretreated group after SE induction. Quantitative analysis of IL-1β and TNF-α **(A,B)** expression levels at 3 h, 24 h, 3 days, and 7 days after SE induction. The protein expression levels of IL-1β and TNF-α **(C,D)** in the SE-asarone were significantly decreased at 3 days following SE. Con, control group; SE, status epilepticus model group; SE-asarone, pretreatment with α-asarone group after SE induction; IL, interleukin; TNF, tumor necrosis factor; ELISA, enzyme-linked immunosorbent assay; *x* ± s, *n* = 5. *p < 0.05, **p < 0.01, vs. Con group; ^#^*p* < 0.05, ^##^*p* < 0.01, vs. SE group.

The proinflammatory cytokine (IL-1β and TNF-α) production in the brain tissue at 3 h, 24 h, 3 days, and 7 days after SE was examined by ELISA. Our results showed that at 3 days following SE, the IL-1β and TNF-α concentration markedly reached a high level in the SE model group compared with the control group (Figures 6A,B). On the other hand, the protein expression levels of IL-1β and TNF-α in the SE-asarone were significantly decreased in the brain region examined (Figures 6C,D).

### Concentration Measurement of α-Asarone in the Brain of Rats after SE *via* HPLC-MS/MS

**Figure 7:**
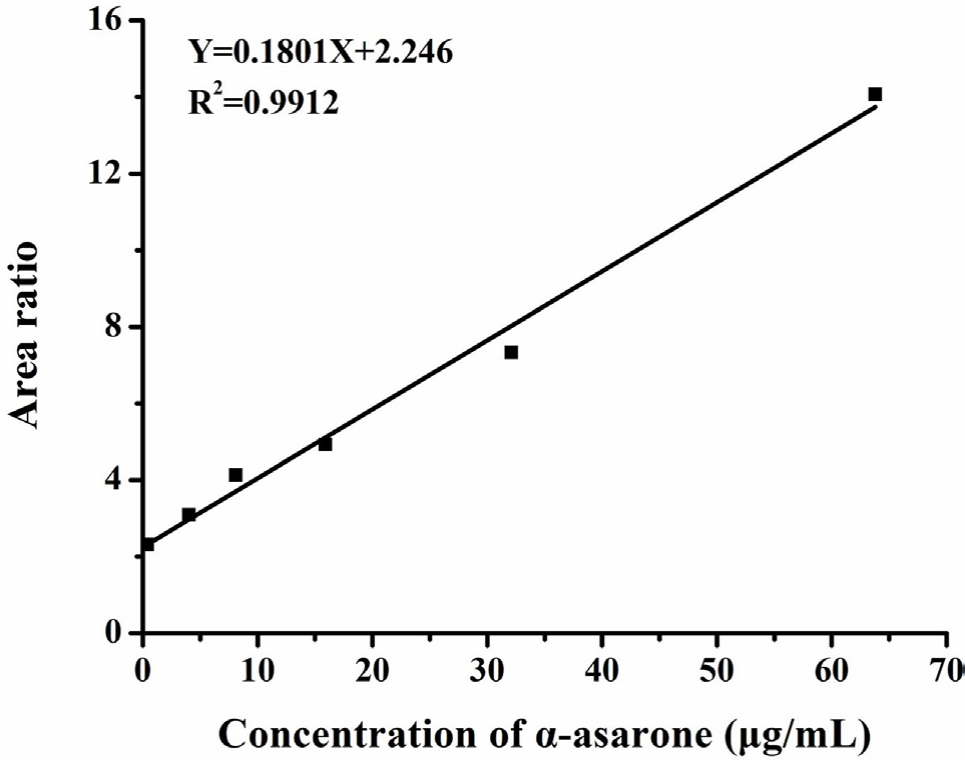
Linear regression of peak area ratios vs. concentrations. *Y* represents the ratio of peak area of α-asarone to peak area of internal standard, *X* represents the concentration of α-asarone solution. The peak area ratios of α-asarone solutions with various concentrations (0.4, 4, 8, 16, 32, and 64 µg/ml) were obtained by HPLC-MS/MS. Then the linear regression was conducted in peak area ratios vs. concentration. The linear fit (*R*^2^) was 0.9912, which implied the effectiveness of linear regression.

Concentrations of α-asarone in the brain of the rats were measured by HPLC-MS/MS. Each peak area of α-asarone in brain tissue extraction solutions and α-asarone solutions with various concentrations was recorded during the measurement. Then linear regression of peak area ratios of α-asarone solutions vs. concentrations was conducted (as shown in Figure 7). The regression equation was as follows:

Y=0.1801X+2.246.

The concentration of α-asarone in brain tissues was calculated by plugging the recorded peak area of α-asarone in brain tissues extraction solution in to the regression equation. And the calculated concentration was 34.9 ± 2.1 μg/ml, which was in line with the range of dose *in vitro*.

### α-Asarone Is Not Toxic to Primary Rat Microglia at Optimal Concentrations

**Figure 8:**
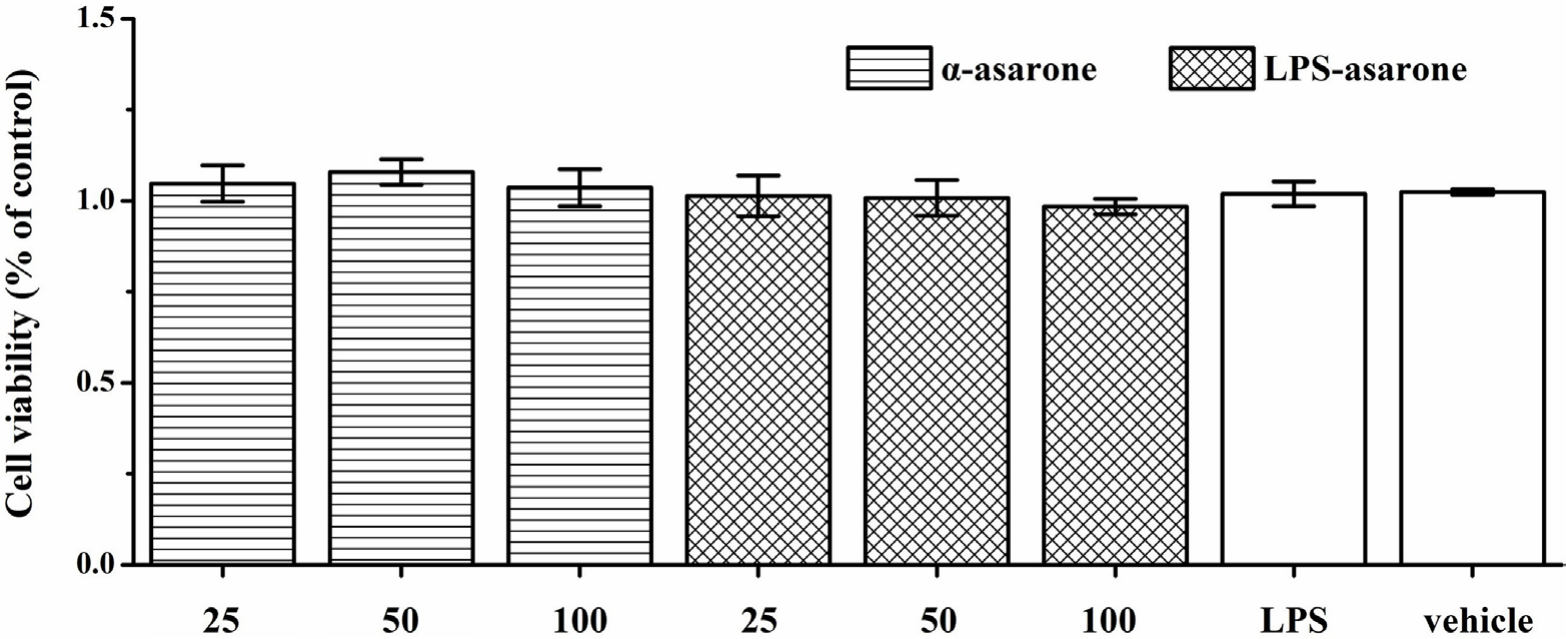
Effect of α-asarone and/or lipopolysaccharide (LPS) on primary rat microglia cell viability as assessed by the MTT assay. Cells were preincubated with α-asarone (25, 50, and 100 µg/ml) and/or LPS (1 µg/ml) for 24 h. The results are shown as the percentages of the treated samples in relation to the control samples, *x* ± s. There was no significant difference in cell viability levels between the microglia in the serum-free Dulbecco’s modified Eagle’s medium-treated controls and the microglia treated with α-asarone (25, 50, and 100 µg/ml) and/or LPS (1 µg/ml).

The cytotoxic effects of α-asarone were examined with an MTT assay by measuring the cell viability of primary rat microglia. Cells were incubated with various concentrations of α-asarone (25, 50, and 100 µg/ml) for 1 h in the presence or absence of LPS (1 µg/ml). There was no significant difference in cell viability between the microglia in the serum-free DMEM-treated controls and the microglia treated with α-asarone or LPS (Figure 8), which confirms that the inhibitory effect of α-asarone on microglia activation was not due to cytotoxicity.

### α-Asarone Alleviates LPS-Stimulated Primary Microglia Activation

**Figure 9:**
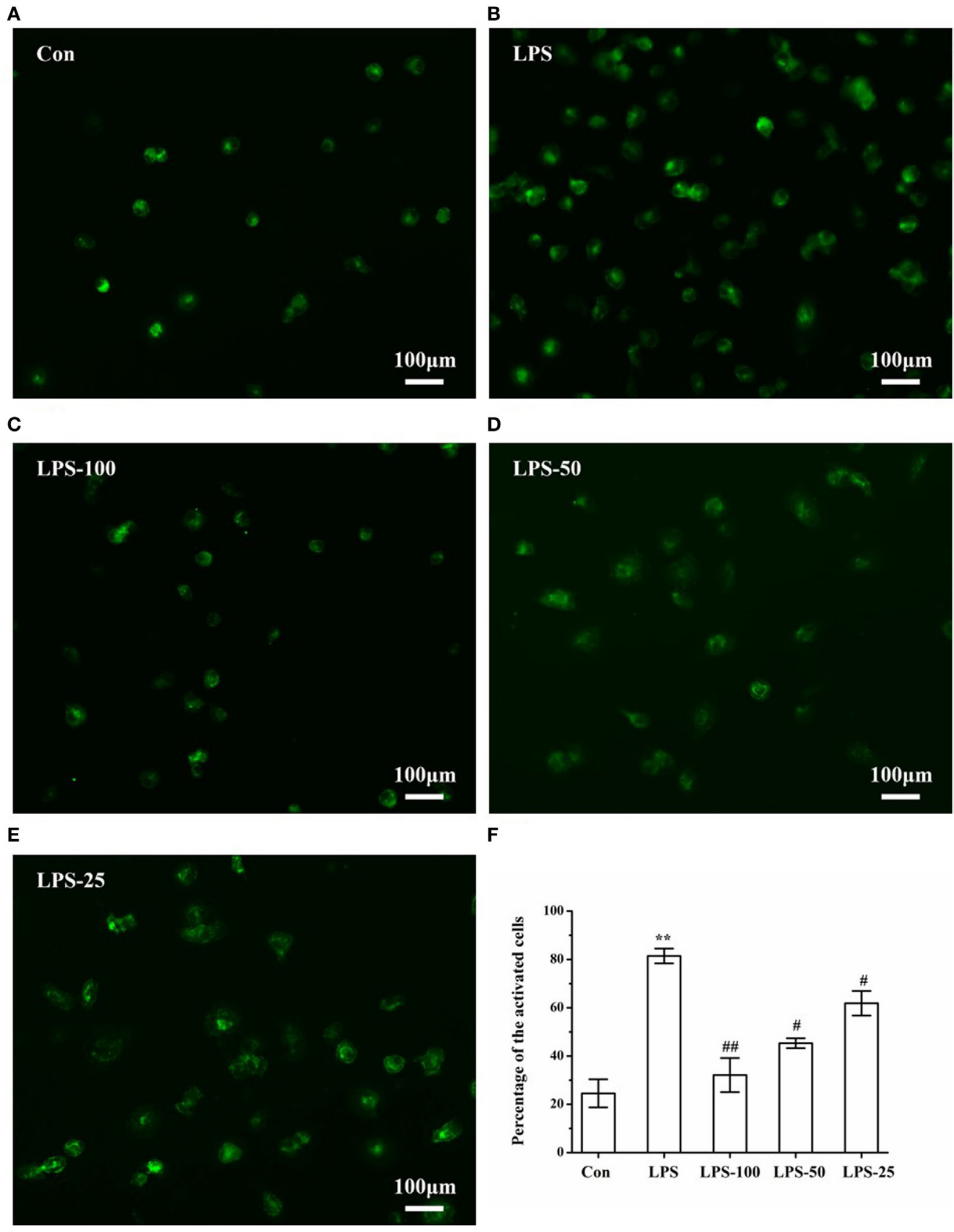
Immunofluorescence images of LPS-induced microglia after α-asarone treatment. Representative images of primary rat microglia cells in the Con group, LPS group, the LPS-100 group, the LPS-50 group, the LPS-25 group **(A–E)**. Cells were pretreated with α-asarone at described concentrations for 1 h. Then, LPS was added to each plate and incubated at 37°C for an additional 3 h. Quantitative analysis of the percentage of activated microglia **(F)**. Con, control group; LPS, lipopolysaccharide-treated model group; LPS-100, LPS-50, LPS-25, microglial pretreatment with different concentrations of α-asarone before LPS treatment (100, 50, 25 µg/ml) group. Scale bar = 100 µm. *x* ± s. *p < 0.05, **p < 0.01, vs. Con group, ^#^*p* < 0.05, ^##^*p* < 0.01, vs. LPS group.

In general, CD-68 only mark activated microglia. Once activated, the cells could be presented clearly under fluorescent. As we can see in Figure 8, after LPS (1 µg/ml) stimulation, cells were activated and pretreatment with α-asarone (25, 50, and 100 µg/ml) could alleviate LPS-stimulated microglia activation (Figures 9C–E). Comparison between groups using ANOVA revealed that the number of activated microglia in the LPS-induced group (Figure 9B) was significantly increased compared to that in the control group (Figure 9A). However, this increase was reversed in the LPS-asarone group (Figure 9F), which means that α-asarone can effectively block the LPS-stimulated microglia activation.

### α-Asarone Alleviates LPS-Stimulated NF-κB Activation in Primary Microglia

**Figure 10:**
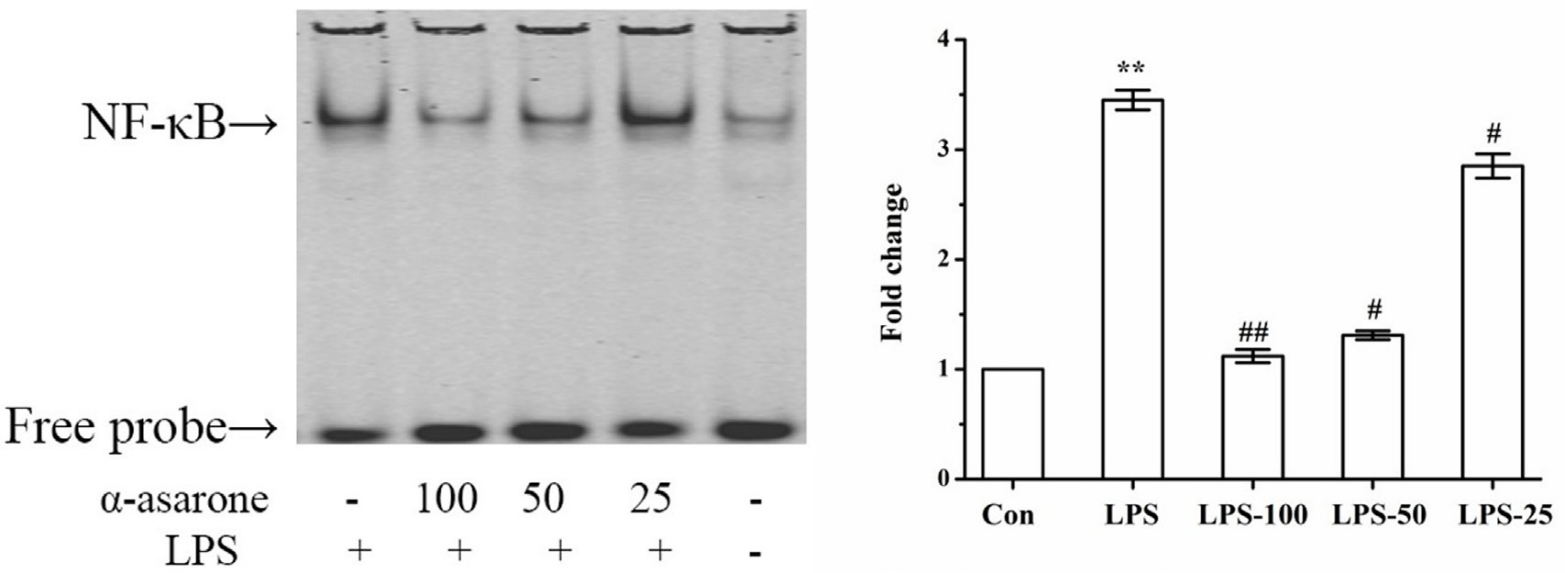
α-Asarone restrained LPS-stimulated NF-κB activation in primary microglia cells. Primary microglia cells were activated with 1 µg/ml LPS but this effect was reversed by the pretreatment with α-asarone (25, 50, and 100 µg/ml) added 1 h before the stimulation. After 3 h, nuclear extracts were isolated for gel shift assay. LPS-stimulated NF-κB DNA binding activity was also suppressed by α-asarone in a dose-dependent manner. Data from triplicate measurements are shown. Con, control group; LPS, lipopolysaccharide-treated model group; LPS-100, LPS-50, LPS-25, microglial pretreatment with different concentrations of α-asarone (100, 50, and 25 µg/ml) group; NF-κB, nuclear factor-κB. *x* ± s. *p < 0.05, **p < 0.01, vs. Con group; ^#^*p* < 0.05, ^##^*p* < 0.01, vs. LPS group.

An increasing number of studies have shown that LPS increases NF-κB subunit activation (36). In order to measure the effect of α-asarone on the DNA binding activity of NF-κB in LPS-induced primary rat microglia, we undertook an EMSA assay. Our results show that NF-κB activation was only markedly induced in the LPS group and that α-asarone significantly reduced LPS-induced NF-κB activation in a dose-dependent manner compared with the control group (Figure 10). These results demonstrate that α-asarone alleviates NF-κB activation in LPS-stimulated primary microglia which might help to explain the mechanism underlying the anti-inflammatory effect of α-asarone.

### α-Asarone Treatment Suppresses the IκB-α and IKK-β Degradation in Primary Microglia Stimulated with LPS

**Figure 11:**
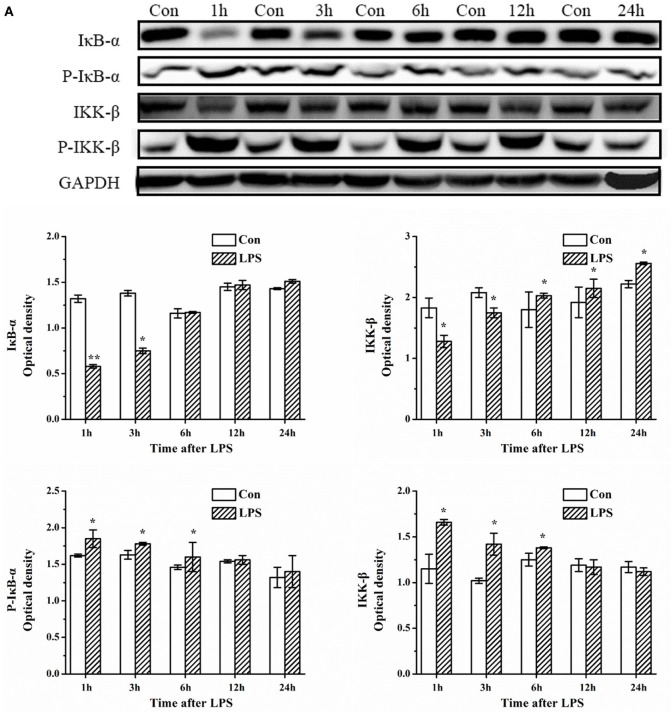

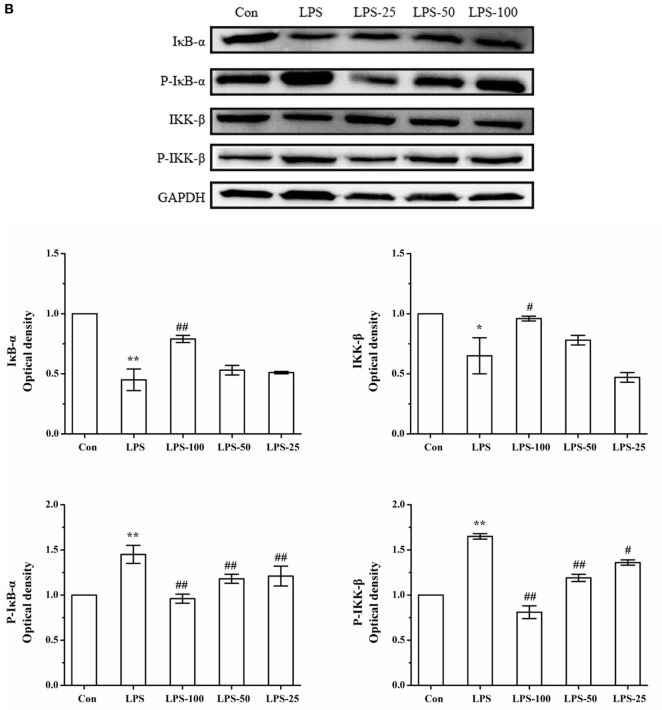
α-Asarone treatment reduces phosphorylation of IκB-α and IKK-β, and thus reduces the available p-IκB-α p-IKK-β kinase for degradation in primary microglia stimulated with LPS. Experiments were performed as previously described. **(A)** LPS exposure induces phosphorylation of IκB-α and IKK-β activation followed by degradation, as assessed by antibodies against phospho-IκB-α, IκB-α, phospho-IKK-β, and IKK-β at different time points. **(B)** After treatment with α-asarone for 1 h, primary rat microglia were stimulated with LPS (1 µg/ml) for 3 h, the protein level were analyzed by Western blot analysis. The p-IκB-α or IκB-α/GAPDH and the p-IKK-β or IKK-β/GAPDH ratio were determined by densitometry analysis. Con, control group; LPS, lipopolysaccharide-treated model group; LPS-100, LPS-50, LPS-25, microglial pretreatment with different concentrations of α-asarone (100, 50, and 25 µg/ml) group; IκB-α, inhibitor kappa B-alpha; IKK-β, I kappaB kinase-β. *x* ± s from three independent experiments. *p < 0.05, **p < 0.01, vs. Con group; ^#^*p* < 0.05, ^##^*p* < 0.01, vs. LPS group.

Next, we focused our attention on the upstream targets of the NF-κB signaling pathway. Western blots showed a significant increase in the phosphorylation levels of IκB-α and IKK-β and an increase in degradation of IκB-α, as well as an increase in IKK-β (kinase) in primary rat microglia at 3 h after LPS exposure which returned to basal levels almost at 24 h later (Figure 11A). However, upon pretreatment with α-asarone, the protein expression levels of phosphorylated IκB-α decreased, the enzyme activation level of IKK-β decreased, followed by a reduction in degradation of the NF-κB/IκB-α dissociation products (p-IκB-α) (Figure 11B).

### Effect of α-Asarone on iNOS and COX-2 Protein Expression in LPS-Stimulated Primary Microglia

**Figure 12:**
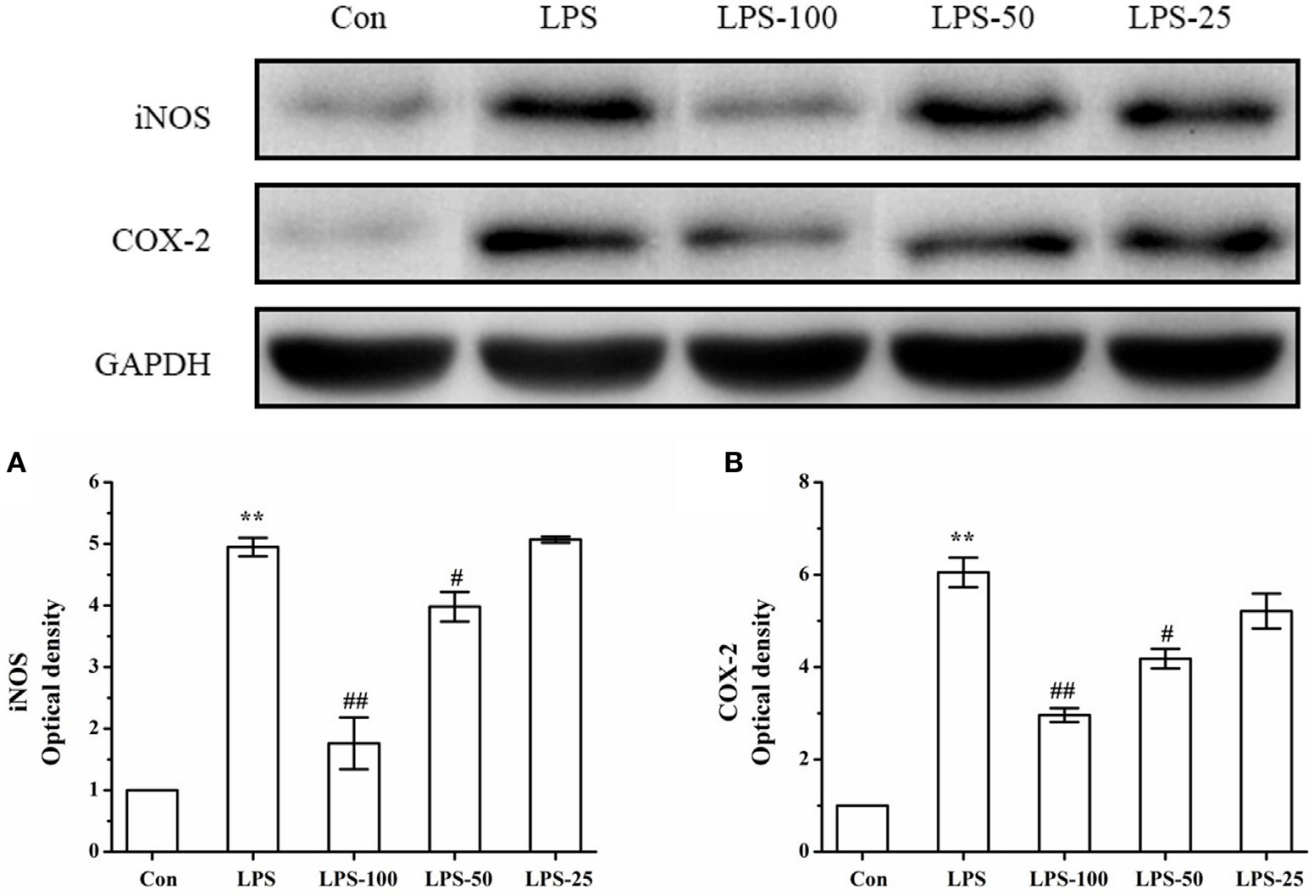
The effect of α-asarone on the iNOS and COX-2 protein expression levels in LPS-stimulated primary microglia. After treatment with α-asarone for 1 h, primary rat microglia were stimulated with LPS (1 µg/ml) for 3 h. The protein level of iNOS **(A)** and COX-2 **(B)** were analyzed by Western blot analysis. Quantitative data are displayed on each panel, respectively. Con, control group; LPS, lipopolysaccharide-treated model group; LPS-100, LPS-50, LPS-25, microglial pretreatment with different concentrations of α-asarone (100, 50, and 25 μg/ml) group; iNOS, including nitric oxide synthase; COX-2, cyclooxygenase-2. *x* ± s from three independent experiments. *p < 0.05, **p < 0.01, vs. Con group; ^#^*p* < 0.05, ^##^*p* < 0.01, vs. LPS group.

To determine the effect of α-asarone on iNOS and COX-2 expression in primary microglia stimulated with LPS, we measured levels of iNOS and COX-2 expression by western blot. As shown in Figure 12, LPS exposure significantly increased iNOS and COX-2 protein expression. Treatment with α-asarone before LPS stimulation significantly decreased significantly decreased protein expression of iNOS and COX-2.

### α-Asarone Attenuates the Protein Expression of IL-1β and TNF-α in LPS-Stimulated Primary Rat Microglia

**Figure 13:**
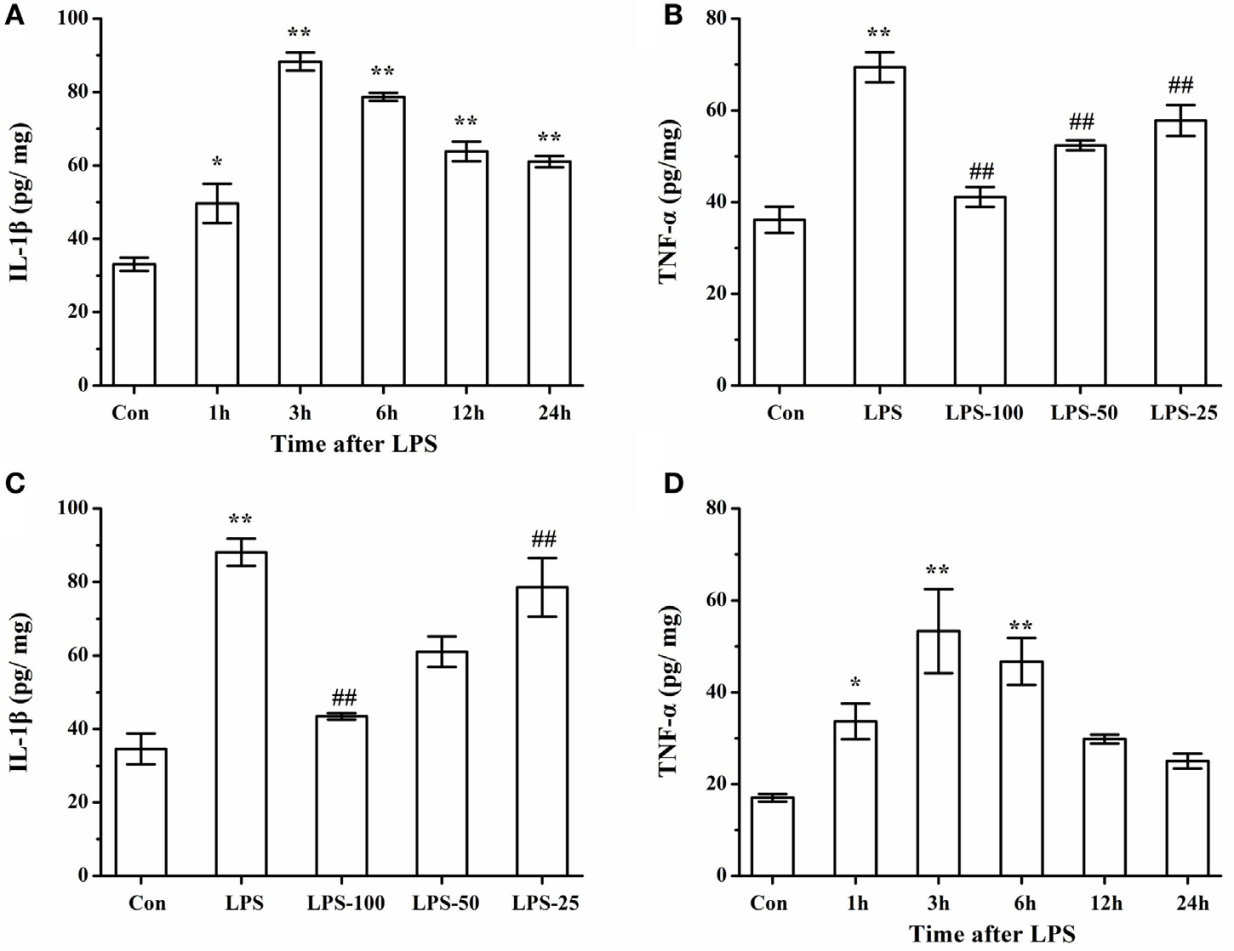
Elisa analysis of IL-1β, TNF-α protein expression LPS simulated cells after α-asarone pretreatment. Quantitative of IL-1β and TNF-α expression levels **(A,B)** at various time points: 1, 3, 6, 12, 24 h. Pretreatment with α-asarone before LPS stimulation significantly decreased IL-1β and TNF-α expression **(C, D)** in the Con, the LPS, the LPS-100, the LPS-50, and the LPS-25 group. Con, control group; LPS: lipopolysaccharide-treated model group; LPS-100, LPS-50, LPS-25, microglia pretreatment with different concentrations of α-asarone (100, 50, and 25 μg/ml) group; IL, interleukin; TNF, tumor-necrosis factor. *x* ± s from three independent experiments. *p < 0.05, **p < 0.01, vs. Con group; ^#^*p* < 0.05, ^##^*p* < 0.01, vs. LPS group.

To determine the effect of α-asarone on the proinflammatory cytokines in primary microglia stimulated with LPS, we measured levels of IL-1β and TNF-α by ELISA. As shown in Figures 13A,B, LPS exposure significantly induced IL-1β and TNF-α expression at different time points, with levels of IL-1β and TNF-α peaking at 3 h. Pretreatment with α-asarone before LPS stimulation significantly decreased IL-1β and TNF-α expression (Figures 13C,D).

## Discussion

Increasing evidence from clinical and experimental studies supports a link between inflammation and epilepsy. Microglial cells perform a significant role in neuroinflammation, and inflammatory mediators such as proinflammation cytokines from microglial cells can amplify brain inflammation (13). Thus, the suppression of microglial activation and alleviation of neuroinflammation might be considered as candidate mechanisms to target during the development of novel AEDs. The present study suggests that α-asarone might attenuate inflammatory activation of microglial cells resulting in the alleviation of brain inflammation, from results obtained using a pilocarpine-induced TLE rat model (*in vivo*) and primary microglial cells (*in vitro*).

Immunohistochemical results showed increased activation of microglial cells marked by staining with CD-68 after pilocarpine-induced SE in rats. Furthermore, the activation of microglial cells after SE induction, increased the levels of NF-κB translocated to the nucleus and the inhibitory protein IκB-α and IKK-β kinase degradation increased upon dissociation from cytosol NF-κB. Moreover, neuroinflammation agents such as IL-1β, TNF-α increased. Meanwhile, iNOS and COX-2 protein expression levels in SE-induced rats were also increased. However, pretreatment with α-asarone alleviated microglial cells activation. In addition, α-asarone alleviates SE-induced cognitive impairment and markedly reduces SRSs in the TLE a rat model. Microglia, the resident immune cells of the CNS, are necessary for normal brain function (37, 38). Once activated, microglia morphology changes from a ramified shape to a round and irregular morphology with shorter axons. Overexcited microglia play a prominent role in the initiation and progression of brain inflammation, involved in the pathogenesis of neurodegenerative disorders, including Alzheimer’s disease, multiple sclerosis and epilepsy (27). Several studies suggested that within the first day of seizures, an early microglia response occurs in the related brain region and the activated microglia could persist for 14 days in a pilocarpine-induced epilepsy model (16, 35). Activated microglia renders developing animals more susceptible to subsequent seizures and seizure-induced inflammatory reaction (39). Our findings are in accordance with previous studies showing that SE caused microglia activation and proliferation at 3 days after SE induction. Clinical evidence demonstrated that activated microglia has been found in the hippocampi specimen of patients with TLE (40) as well as in and around epileptic tubers in tuberous sclerosis patients (41) and in association with epileptic cortical dysplastic lesions (42). Activated microglia is a prominent source of proinflammatory factors such as TNF-α, IL-1β, both of which are neurotoxic and proved to promote hyperexcitability in epilepsy (43). The level of microglial activation has been associated with seizure frequency as well as the length of the epileptic seizure. Furthermore, activated microglia have the ability to decrease seizure threshold which increases further microglial activation (27). This is why the prevention of microglial activation is likely to perform a quintessential function in mitigating learning and memory deficits observed in SE patients.

An increasing body of evidence supports that inflammatory processes within the brain play an important role in the epileptogenesis (9). NF-κB is transcription factor that regulates immune and inflammatory responses. The activity of NF-κB is regulated by its subcellular localization. In most cells, NF-κB is present as a latent, inactive, IκB-bound complex in the cytoplasm, which activation is dependent on the dissociation of the IκB/NF-κB complex by the IκB kinase (IKK). As IKK dissociates NF-κB from IκB-α, it activates NF-κB and induces phosphorylation of the IκB-α protein inhibitors marking these for degradation. Meanwhile, NF-κB dimers translocate to the nucleus and alter the expression of specific genes with NF-κB binding domains (44, 45). NF-κB has been linked with epilepsy for a long time and a large amount of research has explored the direct relationship between NF-κB pathway and epilepsy. Overexpression of NF-κB was found in hippocampal foci of patients with chronic mesial TLE (46). SE-induced in rats exhibited increased expression of NF-κB in neurons (concomitant with microglial activation), an increase in Kainic acid-induced excitotoxicity, a decrease in KA-induced glial cell activation and subsequent increase in expression of proinflammatory genes such as TNF-α and IL-1β (47). Based on these findings, we investigated the NF-κB expression levels in a SE rats model. Our results suggest that there was an increase in protein levels of NF-κB and a decrease of IκB-α and IKK after SE induction. However, pretreatment with α-asarone before SE induction led to a reduction of the cognitive and learning and memory deficit observed in the SE-induced group. Considering the multiple functions of microglia in the CNS and their close association with both inflammation and epilepsy, we also used primary microglia cultures *in vitro* to further verify our hypothesis that α-asarone is a potential neuroprotective agent which can be used in the prevention and treatment of TLE. LPS is an immune stimulant that activates microglia, LPS was proved to increase neuronal excitability by stimulating microglia to release TNF-α and IL-1β (48). Thus, in the present study, we used LPS to activate microglia *in vitro*. Our *in vitro* study produces similar results to the *in vivo* study in terms of changes in protein expression of NF-κB, IκB-α, and IKK. Here, the increase in NF-κB and the decrease in phosphorylated IκB-α, and IKK found in LPS stimulated primary cultured microglia were partially reversed by the α-asarone pretreatment on a dose-dependent basis. These data strongly suggest that α-asarone has an important neuroprotective effect in reducing the activation of the NF-κB pathway. Our results also support the notion that the NF-κB pathway might be a potential mechanism to explore during the development of novel drugs for the treatment of epilepsy.

However, despite the increasing evidence that supports the positive effect of reduced levels of NF-κB in the epileptogenesis, the holistic effect of NF-κB suppression on seizure activity as well as on seizure-induced brain damage is still unclear and controversial (19). Libin found that in kainate-induced seizures and SE, the suppression of NF-κB releasing pathways lead to increased seizure susceptibility and a decreased latency to the onset latency to onset of seizure. These results suggest that NF-κB pathway activation might have a protective function during acute convulsant stimulation (49). Several possibilities may be added to this debate: (1) different epilepsy agents (Kainite and pilocarpine) produced different kinds of SE rat models. Kainite and pilocarpine are both commonly used ways for inducing epilepsy, but they might interfere with NF-κB activation differently. (2) Different inhibitors of NF-κB were applied. A previous study strongly suggested that different regulators of NF-κB pathway may have different effects on cell viability, which may be related to the specific subset of genes targeted by NF-κB once translocated to the nucleus (50). According to Libin, the inhibitors diethyldithiocarbamate and SN50 also could affect other signal pathways besides NF-κB (49). (3) Different timepoints were chosen for observation. In most cases, the activation of NF-κB is transient and cyclical in the presence of a continual inducer (51). Further studies are needed to provide a clear answer in terms of the role of NF-κB activation in epilepsy. In drug-resistant epilepsy, repeated and long-term seizures can cause neuron injury including neuronal necrosis and apoptosis in the hippocampus cells (52). The main cause of neuron apoptosis and hippocampal sclerosis is related to the excitatory activity of seizures and production of toxic neurotransmitters (53). Many studies have reported α-asarone has neural protection activity. Huang et al. found that α-asarone contributes to neural protection by adjusting the balance of excitatory and inhibitory neurotransmitters in the brain, by improving efficiency in the utilization the blood oxygen as well as by regulating the response to oxygen deficit, and by suppressing cell apoptosis (23). α-Asarone can remove effectively free radicals, prevent peroxide formation and reduce the nerve toxicity of NO, therefore conferring protection to brain cells (54). Previous studies showed that LPS-induced microglia activation resulted in an increment of iNOS and COX-2 activation. Our results indicated that the pretreatment with α-asarone significantly attenuated iNOS and COX-2 expression after SE induction (27). This study strongly suggested that α-asarone inhibits the expression of iNOS and COX-2 in LPS-stimulated microglia cells and pilocarpine-induced SE in rat. α-Asarone decreased TNF-α, IL-1β in the rat epilepsy model as well as in activated primary cultured microglia. Mounting evidence suggests that TNF-α, IL-1β from glia cells contribute to the development of epileptic process (55). The contributory role of IL-1β to epileptogenesis has been proved by previous studies. Vezzani et al. first reported that IL-1β upregulated seizure activity in rats by intracerebral application of IL-1β (56). A subsequent study showed that the intracerebral application of IL-1 receptor antagonist (IL-1ra) caused a powerful anticonvulsant effect (57). In the last decades, many investigators were exploring the role of IL-1β in epileptogenesis. Ravizza et al. reported that the frequency of seizures in patients increased with increasing IL-1β levels measured in epileptogenic tissue (58). Gan et al. found that IL-1β was upregulated in a rat model of TLE (46). Our data is in accordance with previous work that supports the fact that IL-1β expression levels were increased in SE-induced rats. Based on these studies, we propose that IL-1β might have an important role in epileptogenesis, rather than being a mere biochemical epiphenomenon. Previous studies reported that IL-1β alters neuronal excitability *via* both “rapid action” and a long-term transcriptional activation (59). In terms of the “rapid action,” IL-1β has been reported to reduce GABA-mediated inhibition and enhance glutamate-mediated excitotoxicity (60, 61). IL-1β also reduces the N-methyl-d-aspartate-induced outward current by activating the P38-mitogen-activated protein (MAPK) pathway (62), which known to be a key mechanism leading to the increase of neuronal excitability. On the other hand, the long-term effects involve the gradual activation of inflammation genes associated with NF-κB at the transcriptional level (59).

The neurotoxic effects of IL-1β in excitotoxicity have been extensively documented. In the same manner, TNF-α has been implicated as a critical mediator affecting seizure susceptibility in animal models. TNF-α level increased rapidly following SE-induction and suppression of TNF-α in the brain is able to decrease epileptic activity (11). Our data also suggest that TNF-α expression was rapidly upregulated within 3 h after SE-induction and reached a peak at 3 days. Nevertheless, it has also been reported that TNF-α expression might perform a neuroprotective function when combined with the TNF-α receptor 2 (63). The α-asarone role in the mitigation of the learning and memory deficits as induced by epilepsy, as presented in our study, might be explained by the α-asarone effects on IL-1β and TNF-α production. These finding might shed some light in terms of the functional effects of these cytokines on neuronal excitability.

## Conclusion

In summary, our results demonstrated that α-asarone could attenuate brain inflammation after SE induction by inhibiting the NF-κB activation pathway in microglia. These findings suggest that α-asarone might be a promising new anti-inflammatory drug for the treatment of epilepsy. In addition, this study provides new insights in terms of the mechanism underlying microglia activation. We propose that α-asarone, which has shown to have neuroprotective activities, might be used for the prevention and treatment of microglia-mediated neuroinflammatory conditions including TLE.

## Availability of Data and Materials

All data generated or analyzed during this study are included in this published article.

## Ethics Statement

All animals were supplied by Chongqing Medical University. All experiments were performed in accordance with the rules of the committee of Experimental Animal Administration of the University and were in accordance with the National Institutes of Guide for Care and Use of Laboratory Animals.

## Author Contributions

QC and J-kM designed the work that led to the submission, acquired data, played an important role in interpreting the results, and approved the final version. YX contributed significantly to analysis and manuscript preparation. HL and XL performed the data analyses and wrote the manuscript and conceived and designed the experiments. QM and JL helped perform the analysis with constructive discussions. CL drafted or revised the manuscript. Y-yH contributed to the conception of the study.

## Conflict of Interest Statement

The authors declare that the research was conducted in the absence of any commercial or financial relationships that could be construed as a potential conflict of interest.

## Abbreviations

TLE, temporal lobe epilepsy; AEDs, antiepileptic drugs; SE, status epilepticus; CNS, central nervous system; LPS, lipopolysaccharide; MTT, 3-(4,5-dimethylthiazole-2-yl)-2,5-diphenyl-tetrazoliumbromide; FBS, fetal bovine serum; SD, Sprague-Dawley; DZP, Diazepam; IκB-α, inhibitor kappa B-alpha; IKK-β, I kappaB kinase-β; IL-1β, interleukin-1β; TNF-α, tumor necrosis factor-α; NF-κB, nuclear factor-κB; MWM, Morris water maze; SRSs, spontaneous recurrent seizures; DMEM, Dulbecco’s modified Eagle’s medium; EMSA, electrophoresis mobility shift assay; iNOS, including nitric oxide synthase; COX-2, cyclooxygenase-2; HPLC-MS/MS, high-performance liquid chromatography-tandem mass spectrometry.
